# Effects of Addition of Systemic Tramadol or Adjunct Tramadol to Lidocaine Used for Intravenous Regional Anesthesia in Patients Undergoing Hand Surgery

**DOI:** 10.1155/2016/9161264

**Published:** 2016-05-30

**Authors:** Abdulkadir Yektaş, Funda Gümüş, Abdulhalim Karayel, Ayşin Alagöl

**Affiliations:** Anesthesiology and Reanimation Clinic, Bagcilar Training and Research Hospital, 34218 Istanbul, Turkey

## Abstract

Intravenous regional anesthesia (IVRA) is used in outpatient hand surgery as an easily applicable and cost-effective technique with clinical advantages. The present study aimed to investigate the effects of addition of systemic tramadol or adjunct tramadol to lidocaine for IVRA in patients undergoing hand surgery. American Society of Anesthesiologists (ASA) I-II patients (*n* = 60) who underwent hand surgery were included. For this purpose, only lidocaine (LDC), lidocaine+adjunct tramadol (LDC+TRA group), or lidocaine+systemic tramadol (LDC+SysTRA group) was administered to the patients for IVRA and the groups were compared in terms of onset and recovery time of sensory and motor blocks, quality of anesthesia, and the degree of intraoperative and postoperative pain. The onset time of sensorial block was significantly shorter in the LDC+TRA group than that in the LDC+SysTRA group. The motor block recovery time was significantly shorter in the LDC+SysTRA group than that in the LDC+TRA and LDC groups. Administration of tramadol as an adjunct showed some clinical benefits by providing a shorter onset time of sensory and motor block, decreasing pain and analgesic requirement, and improving intraoperative conditions during IVRA. It was determined that systemic tramadol administration had no superiority.

## 1. Introduction

Intravenous regional anesthesia (IVRA) is used in outpatient hand surgery as an easily applicable and cost-effective technique with clinical advantages and it is an ideal anesthetic method particularly for short lasting procedures [1]. Although IVRA has a history more than a century old, it has regained importance in the recent years as an effective and safe technique [2]. Nevertheless, IVRA has some disadvantages including anesthetic toxicity, slow-onset, poor muscle relaxation, tourniquet pain, and minimal postoperative pain relief [3]. Providing an ideal anesthesia by overcoming these disadvantages is possible with the addition of some adjunct agents to local anesthetics. These adjunct agents, which are added to the local anesthetics to provide improved block efficacy, decreased tourniquet pain, or prolonged duration of postdeflation analgesia in patients receiving IVRA, include opioids (fentanyl, meperidine, morphine, and sufentanil), tramadol, nonsteroidal anti-inflammatory drugs (NSAIDs; ketorolac, tenoxicam, and acetyl-salicylate), clonidine, and muscle relaxants (atracurium, pancuronium, and mivacurium) [3].

Tramadol, one of the adjunct agents used in IVRA, is a synthetic analgesic and has opioid and nonopioid characteristics. As compared to other opioids, tramadol is advantageous as it has lower side effects and abuse potential [4]. Tramadol has been demonstrated to have local anesthetic effect when administered via intradermal [5] and intravenous [6] routes. In an experimental study conducted on rats, tramadol was shown to block nociception and motor function* in vivo* similar to local anesthetics [7]. Addition of tramadol to mepivacaine has been demonstrated to prolong the duration of brachial plexus block without causing any side effect in patients undergoing forearm and hand surgery [8]. It has been determined that addition of tramadol as an adjunct to bupivacaine for supraclavicular brachial plexus block provides a faster onset of sensorial and motor block and a longer duration of motor block [9].

In light of the abovementioned information, the present study aimed to investigate the effects of addition of tramadol to lidocaine for IVRA in patients undergoing hand surgery. For this purpose, only lidocaine, lidocaine+adjunct tramadol, or lidocaine+systemic tramadol was administered to the patients for IVRA and the groups were compared in terms of onset and recovery times of sensory and motor blocks, quality of anesthesia, and the degree of intraoperative and postoperative pain.

## 2. Methods

### 2.1. Patients

The present study included American Society of Anesthesiologists (ASA) I-II patients (*n* = 60) who were planned to undergo hand surgery. Patients with Raynaud's disease, those with sickle-cell anemia, and those receiving any drug for history of allergy were excluded. Approval of the ethics committee and informed consents of the patients were obtained for the study.

According to a computer-generated randomization list, the patients were divided into three groups, containing 20 subjects in each. In the first group (LDC+TRA group), IVRA was performed with 3 mg/kg lidocaine (10% lidocaine, Aritmal, Biosel, Turkey) plus 50 mg tramadol, which were administered after diluting with saline to 40 mL. While performing IVRA, 30 mL saline was simultaneously administered to the systemic circulation. In the second group (LDC+SysTRA group), IVRA was performed with 3 mg/kg lidocaine, which was diluted with saline to 40 mL. While performing IVRA, 50 mg tramadol diluted with saline to 30 mL was simultaneously administered to the systemic circulation. In the third group (LDC group), IVRA was performed with 3 mg/kg lidocaine, which was diluted with saline to 40 mL. While performing IVRA, 30 mL saline was simultaneously administered to the systemic circulation. All solutions were prepared by resident anesthesiologists, who were blinded to the study, using identical injectors.

### 2.2. Surgical Procedure

The patients received premedication 45 min before the surgery with intramuscular 0.07 mg/kg midazolam and 0.01 mg/kg atropine. Two intravenous cannulas, one into the vein in the dorsal aspect of the hand that would undergo surgery and the other into the vein in the dorsal aspect of the opposite hand, were inserted for crystalloid infusion. The arm that would undergo surgery was elevated for 2 min and Esmarch's bandage was used to control blood flow. A double pneumatic tourniquet was placed around the upper arm and the proximal cuff was inflated to 250 mmHg. The absence of radial artery pulse in the arm isolated from the circulation was confirmed by the disappearance of pulse oximeter waves in the index finger of the same hand. The solutions, which were preprepared according to the groups defined above, were injected to the patients for over 90 s by an anesthesiologist blinded to the contents of drugs.

After the injection, the sensorial block was assessed every 30 s until the initiation of surgery by pinprick test using 22-gauge needle on the radial, ulnar, and median nerve stimulation areas of the hand and of the anterior surface of the arm. Motor function was checked by asking the patient to bring the wrist and finger to extension and flexion and the time of complete motor block was recorded when spontaneous movement was impossible. The time elapsing from the injection of the study drug until the sensorial block was provided in all stimulation areas was recorded as the onset time of sensorial block. Likewise, the time elapsing from the injection of the study drug until achieving the complete motor block was recorded as the onset time of motor block. After achieving complete motor block and sensorial block, the distal tourniquet was inflated to 250 mmHg, the proximal tourniquet was deflated, and the surgical procedure was initiated. Mean arterial pressure (MAP), oxygen saturation (SpO_2_), and heart rate (HR) were monitored during the surgery, before and after tourniquet application, and until disappearance of anesthesia after deflating the tourniquet.

Pain level of the patients was assessed by 10 cm visual analogue scale (VAS; 0: no pain; 10: worst pain imaginable). VAS scores were recorded before and after tourniquet application as well as at 5th, 10th, 15th, 20th, 30th, 40th, and 50th min during the surgery. If the patient had a VAS score of >4 and if required, 1 *μ*g/kg fentanyl was administered for analgesia and the dosage and time were recorded.

The tourniquet was not deflated earlier than 30 min and it was not inflated more than 2 h. Tourniquet deflation after the surgery was performed by periodic deflation technique. The time of sensorial recovery was recorded (the time elapsing from the deflation of tourniquet to the highest pain felt by the patient via pinprick test performed every 30 s in all stimulation areas). The time of motor block recovery (time elapsing from the deflation of tourniquet to the spontaneous movement of the fingers) was also recorded.

The patients were monitored in the postoperative care unit for the first 2 h and then in the observation room for 24 h by anesthesiologists who were blinded to the study. MAP, HR, and SpO_2_ monitoring and VAS measurement were performed at the postoperative 1st, 2nd, 4th, 6th, 12th, and 24th h. The patients with a VAS score of >4 were given 75 mg diclofenac sodium via intramuscular route. Analgesia requirement was recorded as duration and dosage.

The patients were monitored for intraoperative and postoperative complications. In the event of hypotension (systolic arterial pressure < 90 mmHg or a decrease of more than 50 mmHg from the normal value) during the surgery, 5 mg intravenous ephedrine was administered. In case of bradycardia (HR < 50/min), 0.5 mg intravenous atropine was administered. Intravenous 4 mg ondansetron was administered for nausea and vomiting and oxygen was supplied via a facial mask when SpO_2_ decreased by more than 91%.

An anesthesiologist and a surgeon, who were blinded to the content of study drug, assessed the quality of anesthesia at the end of surgery as follows: 4: excellent, patient not complaining; 3: good, patient complaining a little, no need for supplemental analgesic; 2: moderate, patient complaining, need for supplemental analgesic; 1: failed, need for general anesthesia.

### 2.3. Statistical Analysis

The Predictive Analytics Software (PASW) version 18.0 for Windows program (SPSS Inc., Chicago, IL, USA) was used for statistical analysis. Descriptive statistics were expressed as number and percentage for categorical variables and as mean, standard deviation, median, the 25th percentile (Q1: the first quartile), and the 75th percentile (Q3: the third quartile) for numerical variables. For numerical variables, independent multiple group comparisons were performed by Kruskal-Wallis test for nonnormally distributed data and by *t*-test for normally distributed data. Mann-Whitney *U* test with Bonferroni correction was used for subgroup analysis of nonnormally distributed numerical variables. For multiple group comparisons of categorical variables, Chi-square test statistics were used in case the assumption of Chi-square test was met, whereas Fisher's exact test was used in case the assumption of Chi-square test was not met. A *p* value of <0.05 was considered statistically significant.

With the assumption that the difference in the VAS score at the 5th min between the two surgical techniques is 1 and the expected standard deviation for two groups is 0.9, it was estimated to include 20 patients in the groups for which the least difference was expected. The statistical significance level was calculated as 0.015 owing to the presence of 3 groups and with the prediction that repeated measurement analysis would be performed assuming that the Bonferroni correction would be used. The power of the present study was 80% with these calculations.

## 3. Results

General characteristics of the patients are summarized in Table 1. No difference was determined among the groups in terms of age, gender, body weight, height, ASA level, type and duration of surgery, and tourniquet time.

A significant difference was determined among the groups in terms of onset time of sensorial block and recovery time of motor block. The onset time of sensorial block was significantly shorter in the LDC+TRA group than in the LDC+SysTRA group. The motor block recovery time was significantly shorter in the LDC+SysTRA group than in the LDC+TRA and LDC groups (Table 2).

**Figure 1:**
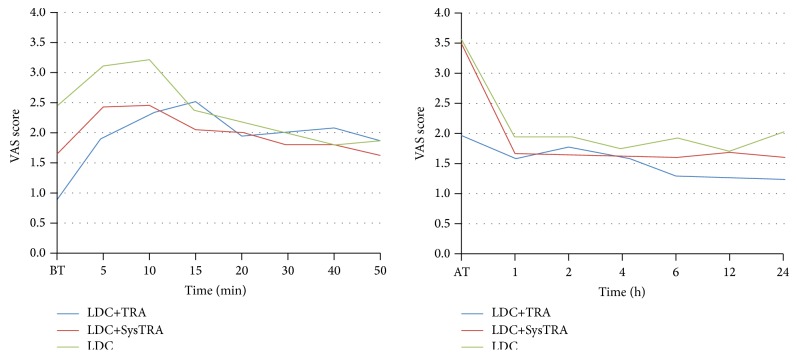
Change in visual analogue scale (VAS) scores in time. BT, before tourniquet; AT, after tourniquet.

The changes in VAS scores in time are illustrated in Figure 1. The VAS scores were observed to be generally lower in the LDC+TRA group. There were significant differences among the groups in terms of VAS scores measured after tourniquet application and at the postoperative 24th h (Table 3).

The patients' need for fentanyl and diclofenac as well as quality of anesthesia, which was assessed by the anesthesiologist and the surgeon, is demonstrated in Table 4. Although it was not found to be significant, the number of patients in need of intraoperative fentanyl and postoperative diclofenac was lower in the LDC+TRA group and the score of the quality of anesthesia was higher in the LDC+TRA and LDC+SysTRA groups.

In the LDC+TRA group, intraoperative adverse events were hypotension (*n* = 2), bradycardia (*n* = 3), nausea (*n* = 1), and shivering (*n* = 1) and postoperative adverse events were nausea (*n* = 1), tinnitus (*n* = 1), and vertigo (*n* = 2). The only postoperative adverse event was vertigo in 1 patient in LDC+SysTRA group. Postoperative shivering (*n* = 1) and metallic taste (*n* = 1) were observed in the LDC group.

## 4. Discussion

Reducing pain and need for analgesics by enhancing the quality of anesthesia is one of the main goals in patients undergoing IVRA. For this purpose, clinical studies have been performed by adding various agents such as dexamethasone [10], midazolam [11], diltiazem [12], dexmedetomidine [13], paracetamol [14], lornoxicam [15, 16], nitroglycerine [17], magnesium [18], and ketamine [19], to the local anesthetic solution and the search for the agent that would provide the most appropriate outcome with the least side effect is ongoing. In the present study, the effects of addition of tramadol to lidocaine were evaluated. In addition, the effects of addition of adjunct or systemic tramadol to lidocaine were compared.

In the present study, the absence of difference among the three patient groups in terms of demographic characteristics such as age, gender, body weight, and height, as well as ASA level, type and duration of surgery, and tourniquet time suggested that the groups were comparable in terms of other parameters. Onset times of sensorial and motor blocks were found to be shorter in the group that received tramadol as an adjunct in IVRA application than in the other two groups; however, the difference was significant only between the LDC+TRA and LDC+SysTRA groups in terms of onset time of sensorial block. In general, VAS scores tended to be lower when tramadol was added to lidocaine. However, statistical significance was determined after tourniquet application and at the postoperative 24th h. With regard to need for analgesics, the number of patients in need of intraoperative fentanyl and postoperative diclofenac was lower in the LDC+TRA group and the scores of quality of anesthesia were higher in the two groups that received tramadol than in the group that received only lidocaine; however, the differences were not statistically significant.

In the literature, studies on the addition of tramadol to lidocaine during IVRA have reported different results. Acalovschi et al. [20] conducted a study in voluntary medical students (*n* = 60) and concluded that a solution including 100 mg tramadol alone had no local anesthetic effect for IVRA. Nevertheless, when administered together with lidocaine, tramadol modifies its effect and shortens the onset time of sensory block in IVRA. Aslan et al. [21] investigated the effect of addition of morphine or tramadol (1.5 mg/kg) to lidocaine for IVRA in 90 patients undergoing hand and forearm surgery. They concluded that the addition of morphine or tramadol to lidocaine enhanced the levels of sensorial block and postoperative analgesia with no effect on tourniquet pain, quality of motor block, duration of analgesia, and analgesic consumption. Alayurt et al. [22] conducted a study in 60 patients undergoing hand surgery and reported that the addition of 100 mg tramadol to lidocaine for IVRA increased the quality of anesthesia, reduced the onset of the sensory block, delayed the onset time of tourniquet pain, and decreased the intraoperative consumption of opioid; however, it had no effect on the postoperative pain. Langlois et al. [23] investigated the effect of addition of 100 mg tramadol to lidocaine for IVRA in 30 patients undergoing carpal tunnel decompression. They reported that pain scales and analgesic request did not differ at any of the time periods studied; accordingly, efficacy of tramadol and lidocaine combination was concluded not to be higher than lidocaine alone. In their study on 54 patients undergoing upper limb surgery, Tan et al. [24] reported that the addition of 50 mg tramadol to lidocaine for IVRA appeared to be helpful in enhancing the quality of anesthesia and observed that higher number of patients had faster onset of sensory and motor block in the group that received tramadol, although the difference was not significant. Moreover, the pain score 30 min after tourniquet inflation and after changing over to the distal tourniquet was significantly lower in the lidocaine+tramadol group than in the group that received lidocaine alone. The reason for different results obtained in different studies may be the heterogeneous patient groups as well as different tramadol doses used in the studies. In their study on 60 ASA I-II patients who were planned to undergo hand surgery, Siddiqui et al. [25] compared the effects of addition of two different doses of tramadol (50 mg versus 100 mg) to lidocaine. They reported that tramadol 100 mg shortened the onset of sensory block, increased the tourniquet tolerance, and improved the perioperative analgesia and thereby concluded that addition of tramadol 100 mg to lidocaine is useful for IVRA.

In their review, Flamer and Peng [26] compared the local anesthetics and adjunct substances used for IVRA in terms of intraoperative efficacy and postoperative outcomes and reported an acceleration in the onset of sensory block, tourniquet tolerance but inconsistent postoperative benefits, and increased risk of minor side effects with the use of tramadol. It has been reported that intravenous tramadol administration reduces postoperative pain and shivering [27]. In the present study, postoperative shivering was observed in 1 patient and metallic taste was observed in 1 patient in the group that received only lidocaine. In the group that received tramadol as an adjunct, hypotension (*n* = 2), bradycardia (*n* = 3), nausea (*n* = 1), and shivering (*n* = 1) were noted intraoperatively, whereas nausea (*n* = 1), tinnitus (*n* = 1), and vertigo (*n* = 2) were observed postoperatively. In the group that received systemic tramadol, only one patient developed postoperative vertigo.

In conclusion, administration of tramadol as an adjunct showed some clinical benefits by providing a shorter onset time of sensory and motor block, decreasing pain and analgesic requirement, and improving intraoperative conditions during IVRA. The visual analogue scale scores were observed to be generally lower in the LDC+TRA group, and the score of the quality of anesthesia was higher in the LDC+TRA and LDC+SysTRA groups.

## Figures and Tables

**Table 1:** General characteristics of the patients.

	Groups			*p*
	LDC+TRA *n* = 20	LDC+SysTRA *n* = 20	LDC *n* = 20	
Age, year	36.55 ± 11.82	41.65 ± 12.47	44.1 ± 13.09	0.063
Gender				
Male	9 (45.0)	14 (70.0)	13 (65.0)	0.233
Female	11 (55.0)	6 (30.0)	7 (35.0)	
Body weight, kg	74.25 ± 15.78	78.6 ± 14.01	77 ± 16.52	0.594
Height, cm	167.95 ± 11.76	172.9 ± 10.7	169.8 ± 10.63	0.605
ASA				
I	15 (75.0)	17 (85.0)	15 (75.0)	0.675
II	5 (25.0)	3 (15.0)	5 (25.0)	
Type of surgery				
Carpal tunnel	5 (25.0)	7 (35.0)	8 (40.0)	0.592
Guyon's channel	6 (30.0)	6 (30.0)	6 (30.0)	1.000
Tendon repair	8 (40.0)	7 (35.0)	6 (30.0)	0.803
Duration of surgery, min	27 (20–40)	25 (20–32.5)	25 (20–33)	0.912
Tourniquet time, min	38 (32.5–55)	42 (36.5–47)	35 (30–43.5)	0.104

ASA, American Society of Anesthesiologists.

Values are presented as mean ± standard deviation, number (%), or median (Q1–Q3).

**Table 2:** Onset and recovery times for sensorial block and motor block.

	Groups			*p*
	LDC+TRA *n* = 20	LDC+SysTRA *n* = 20	LDC *n* = 20	
SB onset time, s	115 (60–124)^b^	180 (120–205)^a^	120 (80–180)	**0.015**
SB recovery time, s	120 (60–180)	89.5 (60–90)	90 (84–120)	0.071
MB onset time, s	120 (90–211.5)	210 (180–245)	210 (180–240)	0.166
MB recovery time, s	120 (69.5–180)^b^	30 (30–60)^ac^	115 (75–170)^b^	**<0.001**

SB, sensorial block; MB, motor block.

Values are presented as median (Q1–Q3).

^a^Different from the LDC+TRA group; ^b^different from the LDC+SysTRA group; ^c^different from the LDC group (*p* < 0.017 with Bonferroni correction).

**Table 3:** Visual analogue scale scores at different times.

Measuring time	Groups			*p*
	LDC+TRA *n* = 20	LDC+SysTRA *n* = 20	LDC *n* = 20	
Before tourniquet	1 (1-1)	1 (1-1)	1 (1–3.5)	0.058
After tourniquet	1.5 (0–4)^bc^	4 (2–5)^a^	3 (2–5.5)^a^	**0.041**
Intraoperative 5th min	1.5 (0.5–3)	2 (1.5–3)	3 (1.5–4.5)	0.103
Intraoperative 10th min	2 (0–4)	2 (2-3)	2 (2–5)	0.409
Intraoperative 15th min	2 (0–3.5)	2 (1.5–2)	2 (2-3)	0.761
Intraoperative 20th min	2 (0.5–2)	2 (2-2)	2 (2-2)	0.895
Intraoperative 30th min	2 (0.5–2)	2 (2-2)	2 (2-2)	0.852
Intraoperative 40th min	2 (1-2)	2 (2-2)	2 (2-2)	0.984
Intraoperative 50th min	2 (0.5–2)	2 (1.5–2)	2 (2-2)	0.739
Postoperative 1st h	2 (0–2)	2 (1.5–2)	2 (2-2)	0.387
Postoperative 2nd h	2 (0.5–2)	2 (1.5–2)	2 (2-2)	0.434
Postoperative 4th h	2 (1-2)	2 (1.5–2)	2 (2-2)	0.692
Postoperative 6th h	2 (0.5–2)	2 (1.5–2)	2 (2-2)	0.092
Postoperative 12th h	1.5 (0.5–2)	2 (2-2)	2 (2-2)	0.081
Postoperative 24th h	1.5 (0.5–2)^c^	2 (1.5–2)	2 (2-2)^a^	**0.025**

Values are presented as median (Q1–Q3).

^a^Different from the LDC+TRA group; ^b^different from the LDC+SysTRA group; ^c^different from the LDC group (*p* < 0.017 with Bonferroni correction).

**Table 4:** Patients' need for analgesics and evaluation of quality of anesthesia.

	Groups			*p*
	LDC+TRA *n* = 20	LDC+SysTRA *n* = 20	LDC *n* = 20	
Fentanyl				
Patients in need	6 (30.0)	12 (60.0)	13 (65.0)	0.057
Time of initial need, min	0 (0–5)	5 (0–7.5)	5 (0–10)	0.265
Intraoperative consumption, *μ*g	0 (0–62.5)	50 (0–75)	50 (0–75)	0.142
Diclofenac sodium				
Patients in need	15 (75.0)	18 (90.0)	20 (100.0)	0.055
Time of initial need, min	24 (1.5–420)	360 (120–420)	360 (240–360)	0.071
Total consumption, mg	75 (37.5–150)	75 (75-75)	75 (75-75)	0.801
Score of the quality of anesthesia				
Assessed by the anesthesiologist	4 (3-4)	4 (3-4)	3.5 (2.5–4)	0.334
Assessed by the surgeon	4 (3.5–4)	4 (3-4)	3.5 (2–4)	0.201

Values are presented as median (Q1–Q3) or number (%).

## References

[1] Chan V. W. S., Peng P. W. H., Kaszas Z. (2001). A comparative study of general anesthesia, intravenous regional anesthesia, and axillary block for outpatient hand surgery: clinical outcome and cost analysis. *Anesthesia and Analgesia*.

[2] dos Reis A. (2008). Intravenous regional anesthesia—first century (1908–2008). Beggining, development, and current status. *Revista Brasileira de Anestesiologia*.

[3] Choyce A., Peng P. (2002). A systematic review of adjuncts for intravenous regional anesthesia for surgical procedures. *Canadian Journal of Anesthesia*.

[4] Vazzana M., Andreani T., Fangueiro J. (2015). Tramadol hydrochloride: pharmacokinetics, pharmacodynamics, adverse side effects, co-administration of drugs and new drug delivery systems. *Biomedicine and Pharmacotherapy*.

[5] Pang W.-W., Mok M. S., Chang D.-P., Huang M.-H. (1998). Local anesthetic effect of tramadol, metoclopramide, and lidocaine following intradermal injection. *Regional Anesthesia and Pain Medicine*.

[6] Pang W.-W., Huang P.-Y., Chang D.-P., Huang M.-H. (1999). The peripheral analgesic effect of tramadol in reducing propofol injection pain: a comparison with lidocaine. *Regional Anesthesia and Pain Medicine*.

[7] Sousa A. M., Ashmawi H. A., Costa L. S., Posso I. P., Slullitel A. (2012). Percutaneous sciatic nerve block with tramadol induces analgesia and motor blockade in two animal pain models. *Brazilian Journal of Medical and Biological Research*.

[8] Kapral S., Gollmann G., Waltl B. (1999). Tramadol added to mepivacaine prolongs the duration of an axillary brachial plexus blockade. *Anesthesia and Analgesia*.

[9] Nagpal V., Rana S., Singh J., Chaudhary S. K. (2015). Comparative study of systemically and perineurally administered tramadol as an adjunct for supraclavicular brachial plexus block. *Journal of Anaesthesiology Clinical Pharmacology*.

[10] Bigat Z., Boztug N., Hadimioglu N., Cete N., Coskunfirat N., Ertok E. (2006). Does dexamethasone improve the quality of intravenous regional anesthesia and analgesia? A randomized, controlled clinical study. *Anesthesia and Analgesia*.

[11] Kashefi P., Montazeri K., Honarmand A., Safavi M., Hosseini H. M. (2011). The analgesic effect of midazolam when added to lidocaine for intravenous regional anaesthesia. *Journal of Research in Medical Sciences*.

[12] Khanna P., Mohan V. K., Sunder R. A. (2013). Efficacy of diltiazem as an adjunct to lignocaine in intravenous regional anesthesia. *Saudi Journal of Anaesthesia*.

[13] Memiş D., Turan A., Karamanlioğlu B., Pamukçu Z., Kurt I. (2004). Adding dexmedetomidine to lidocaine for intravenous regional anesthesia. *Anesthesia and Analgesia*.

[14] Sen H., Kulahci Y., Bicerer E., Ozkan S., Dagl G., Turan A. (2009). The analgesic effect of paracetamol when added to lidocaine for intravenous regional anesthesia. *Anesthesia and Analgesia*.

[15] Sen S., Ugur B., Aydin O. N., Ogurlu M., Gezer E., Savk O. (2006). The analgesic effect of lornoxicam when added to lidocaine for intravenous regional anaesthesia. *British Journal of Anaesthesia*.

[16] Sertoz N., Kocaoglu N., Ayanoğlu H. Ö. (2013). Comparison of lornoxicam and fentanyl when added to lidocaine in intravenous regional anesthesia. *Brazilian Journal of Anesthesiology*.

[17] Sen S., Ugur B., Aydin O. N., Ogurlu M., Gursoy F., Savk O. (2006). The analgesic effect of nitroglycerin added to lidocaine on intravenous regional anesthesia. *Anesthesia and Analgesia*.

[18] Turan A., Memiş D., Karamanlioğlu B., Güler T., Pamukçu Z. (2005). Intravenous regional anesthesia using lidocaine and magnesium. *Anesthesia and Analgesia*.

[19] Viscomi C. M., Friend A., Parker C., Murphy T., Yarnell M. (2009). Ketamine as an adjuvant in lidocaine intravenous regional anesthesia: a randomized, double-blind, systemic control trial. *Regional Anesthesia and Pain Medicine*.

[20] Acalovschi I., Cristea T., Margarit S., Gavrus R. (2001). Tramadol added to lidocaine for intravenous regional anesthesia. *Anesthesia and Analgesia*.

[21] Aslan B., Izdeş S., Kesimci E., Gümüs T., Kanbak O. (2009). Comparison of the effects of lidocaine, lidocaine plus tramadol and lidocaine plus morphine for intravenous regional anesthesia. *Agri*.

[22] Alayurt S., Memis D., Pamukcu Z. (2004). The addition of sufentanil, tramadol or clonidine to lignocaine for intravenous regional anaesthesia. *Anaesthesia and Intensive Care*.

[23] Langlois G., Estèbe J.-P., Gentili M. E., Kerdilès L., Mouilleron P., Ecoffey C. (2002). The addition of tramadol to lidocaine does not reduce tourniquet and postoperative pain during iv regional anesthesia. *Canadian Journal of Anesthesia*.

[24] Tan S. M., Pay L. L., Chan S. T. (2001). Intravenous regional anaesthesia using lignocaine and tramadol. *Annals of the Academy of Medicine Singapore*.

[25] Siddiqui A. K., Mowafi H. A., Al-Ghamdi A. M., Ismail S. A., AbuZeid H. A. (2008). Tramadol as an adjuvant to intravenous regional anesthesia with lignocaine. *Saudi Medical Journal*.

[26] Flamer D., Peng P. W. H. (2011). Intravenous regional anesthesia: a review of common local anesthetic options and the use of opioids and muscle relaxants as adjuncts. *Local and Regional Anesthesia*.

[27] Grond S., Sablotzki A. (2004). Clinical pharmacology of tramadol. *Clinical Pharmacokinetics*.

